# Low Serum Albumin level as a Predictor of Hemorrhage Transformation after Intravenous Thrombolysis in Ischemic Stroke Patients

**DOI:** 10.1038/s41598-017-06802-y

**Published:** 2017-08-10

**Authors:** Ruiwen Che, Xiaoqin Huang, Wenbo Zhao, Fang Jiang, Longfei Wu, Zhen Zhang, Tingting Bian, Qingfeng Ma, Zhipeng Yu, Qian Zhang, Kai Dong, Haiqing Song, Xunming Ji

**Affiliations:** 1 Department of Neurology, Xuan Wu Hospital, Capital Medical University, Beijing, 100053 China; 2 Department of Neurosurgery, Xuan Wu Hospital, Capital Medical University, Beijing, 100053 China

## Abstract

Serum albumin levels has been shown to predict outcome in ischemic stroke patients. We aimed to investigate the relationship between serum albumin levels and hemorrhagic transformation (HT) after intravenous thrombolysis (IVT) in patients with acute stroke. 428 patients receiving intravenous rt-PA therapy were included from 2013 to 2016 and were categorized into two groups: low level (<35 mmol/L) and normal level (35–55 mmol/L) group. Demographic, clinical and laboratory information, HT and functional outcomes were analyzed. Hemorrhagic transformation was comfirmed by CT scan or MRI within 7 days. The functional outcome was measured by modified Barthel Index and modified Rankin Scale (mRS) at 7 days and 90 days. Patients with lower albumin had significantly higher risk of HT (15.3% vs. 4.2%, P = 0.002) and sICH (6.2% vs. 1.4%, P = 0.03) than those with normal level of albumin. In univariate analysis for HT, atrial fibrillation and level of albumin were identified as significant factors (P < 0.001, P = 0.001 respectively). On multivariate logistic regression analysis, serum albumin level remained independent predictor of HT (OR = 4.369, 95% CI = 1.626–11.742, P = 0.003). No significantly difference were found in the clinical outcome at 7 days and 90 days between two groups ﻿(*P* > 0.05). Low level of serum albumin within 24 hours may be an independent predictor of post-thrombolytic HT.

## Introduction

Intravenous (IV) recombinant tissue plasminogen activator (rt-PA) is thought to be the only method of clinical management of acute ischemic stroke (AIS). Hemorrhagic transformation (HT) is the main complication, which can worsen the clinical course and outcome of ischemic stroke. The mechanism of HT after thrombolysis is complex.

Serum albumin, which is synthesized only in liver, is a biochemical marker of nutritional status. In experimental studies, serum albumin shows its neuroprotective function by hemodilution and reducing oxidative stress^1–3^. Previous studies have demonstrated that albumin levels is associated with increased stroke risk and poor outcome in acute ischemic stroke^4, 5^. For IVT-treated AIS patients, the relationship between albumin levels and hemorrhagic transformation is unknown. The aim of this study was therefore to investigate (1) the correlation between serum albumin levels with hemorrhagic transformation and (2) clinical outcome for AIS in patients treated with IVT.

## Results

**Figure 1:**
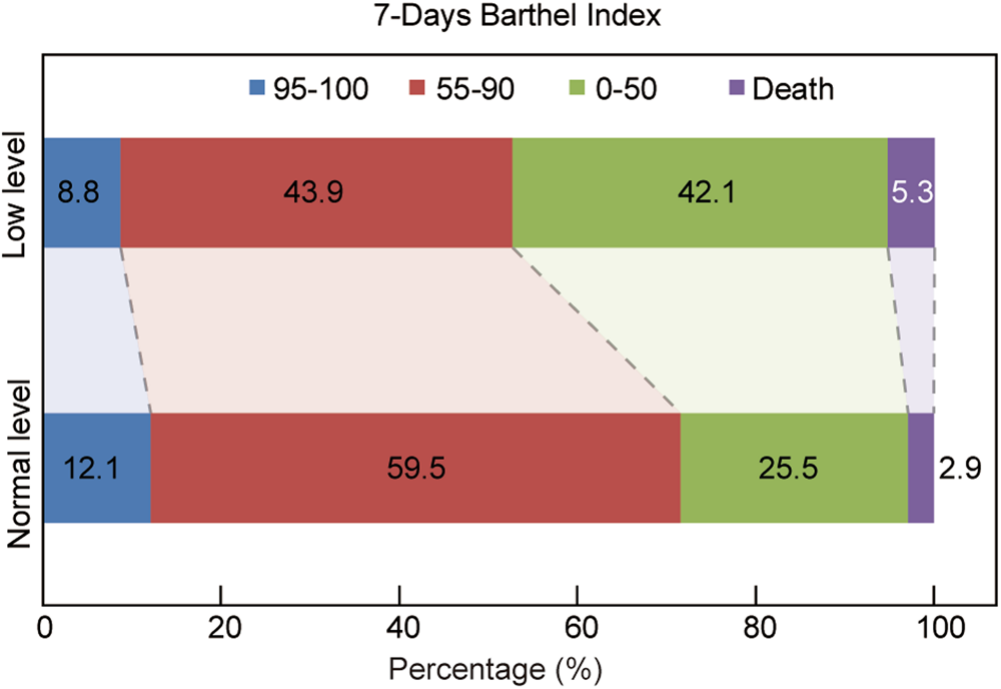
The raw distribution of scores on the Barthel Index at 7 days in the group with low level of albumin (less than 35 mmol/L) and the group with normal level of albumin (35–55 mmol/L). Categorizing patients into 4 groups according to Barthel Index: score 95–100 indicating no symptoms, 55–99 slight-to-moderate disability, 0–50 severe disability. In this picture, fewer people scored 55–100 were found in low level of serum albumin, but there is no statistically significant difference between two groups (*P* = 0.061).

A total of 428 consecutive AIS patients receiving IV rt-PA within 4.5 h of symptom onset were identified from January 2013 to November 2016. Demographic and clinical characteristics of each group are shown in Table 1. The median age of the patients was 61 (27–88) years old, and 316 patients (73.83%) were men. The median onset-to-needle time was 190.0 (IQR 137.0–254.4) minutes and the median initial NIHSS score was 5.0 (IQR 3.0–10.0). The median serum albumin level was 39.13 (IQR 36.25–41.58 mmol/L). Sixty-five (15.2%) patients show a low level of serum albumin ( <35 mmol/L), 363 (84.8%) the patients have a normal serum albumin level (35–55 mmol/L). No patients with a high level of serum albumin ( >55 mmol/L). So all patients were categorized into two groups according to level of serum albumin. Those patients with low level of serum albumin were elderly (66 vs. 61, *P* = 0.001) and a little higher in INR (1.06 vs. 1.04, *P* = 0.014) than patients with normal level albumin. More statins were used for the patients with normal level of albumin (85.5% vs. 73.6%, *P* < 0.05). Twenty-four patients (5.6%) developed HT within 7 days (see Table 1). More anticoagulation medicines and statins were used in non-hemorrhagic transformation patients (*P* < 0.05). No significant differences in the antihypertensive drugs and antidiabetic drugs (*P* > 0.05) (see Table 2). For the clinical outcomes, no significant differences were observed in the median Barthel Index (60.0 vs. 75.0, *P* = 0.061) between normal level and low level of albumin group at 7 days (see Fig. 1). There was also no difference between two levels of serum albumin groups in NIHSS score at 7 days (3 vs. 3, *P* = 0.53). The proportion of patients with favorable outcome (mRS < 3) shows no difference at 90 days (67.5% vs. 64.7%, *P* = 0.698).

**Table 1:** Baseline Characteristics and Outcome Measures of Stroke Patients Receiving Different levels of Albumin in Serum.

	Low level of albumin group (n = 65)	Normal level of albumin group (n = 363)	*P* Value
Age, y, median (IQR)	66 (59–73)	61 (53–67)	0.002*
Male sex, n(%)	43 (66.2)	273 (75.2)	0.13
Medical history			
Hypertension, n(%)	246 (67.8)	37 (59.7)	0.21
Diabetes mellitus, n(%)	117 (32.2)	20 (30.8)	0.82
Atrial Fibrillation, n(%)	46 (12.7)	8 (12.9)	0.96
Coronary artery disease, n(%)	64 (17.6)	12 (19.4)	0.74
Current Smoking, n(%)	92 (25.4)	12 (19.4)	0.31
Current Alcoholism, n(%)	44 (12.2)	9 (14.5)	0.61
Previous stroke, n(%)	92 (25.4)	12 (19.4)	0.31
OCSP			0.916
LACI, n(%)	14 (25.9)	97 (27.2)	--
TACI, n(%)	7 (13.0)	35 (9.8)	--
PACI, n(%)	23 (42.6)	158 (44.4)	--
POCI, n(%)	10 (13.2)	66 (18.5)	--
Laboratory measures			
Glucose, mmol/L, median (IQR)	5.69 (4.95–8.02)	5.75 (4.97–7.26)	1.00
LDL-cholesterol, mmol/L, median (IQR)	2.46 (1.94–3.02)	2.74 (2.21–3.25)	0.06
INR, Median (IQR)	1.06 (1.02–1.12)	1.04 (0.98–1.1)	0.014*
Anticoagulation and statins therapy before admisson			
antiplatelet drugs, n(%)	44 (12.2)	9 (14.5)	0.61
anticoagulants, n(%)	4 (1.1)	0 (0)	1.00
statins, n(%)	4 (6.6)	33 (3.1)	0.67
Previous use of antihypertensive drugs, n(%)	24 (39.3)	132 (36.5)	0.67
Antidiabetic therapy before enrollment, n(%)	15 (24.2)	75 (20.8)	0.54
Antiplatelet therapy after admission			
Aspirin 100 mg/d, n(%)	16 (29.6)	131 (38.2)	0.23
Clopidogrel 75 mg/d, n(%)	12 (19.0)	48 (13.3)	0.23
Aspirin 100 mg/d + Clopidogrel 75 mg/d, n(%)	21 (38.9)	119 (34.7)	0.55
Warfarin, n(%)	0 (0)	16 (4.5)	0.22
Statins			
Rosuvastatin, n(%)	9 (16.7)	20 (5.9)	0.019
Atorvastatin, n(%)	37 (56.9)	290 (79.9)	< 0.001
Antihypertensive drugs, n(%)	8 (11.1)	64 (18.9)	0.72
Antidiabetic drugs, n(%)	7 (10.8)	43 (11.8)	0.89
NIHSS on admission, median (IQR)	5 (3–11)	5 (3–10)	0.89
DNT, min, median (IQR)	55.5 (42–100)	54.5 (43–101)	1.00
ONT, min, median (IQR)	182.5 (125.75–255.75)	194 (140–254)	0.54
Treatment in extent time-window 3–4.5 h, n(%)	13 (21.0)	139 (41.4)	0.002*
Systolic blood pressure, mmHg, median (IQR)	140 (125–165)	140 (137–161)	0.24
Hemorrhagic transformation, n(%)	9 (15.3)	15 (4.2)	0.002*
Symptom intracranial hemorrhage, n(%)	5 (6.2)	4 (1.4)	0.03*
NIHSS at 24 h, median (IQR)	3 (1–8)	3 (1–9)	0.90
NIHSS at 7 days, (median (IQR)	2 (0–5)	1.5 (0–5.75)	0.53
Barthel Index at 7 days, median (IQR)	75 (50.0–90.0)	60.0 (25.0–87.5)	0.061
mRS at 7 days, median (IQR)	2 (1–4)	2 (0–4)	0.84
mRS at 90 days, median (IQR)	2 (0–3)	1 (0–3)	0.698
favorable outcome at 90 days, n(%)	33 (64.7)	195 (67.5)	0.698

P < 0.05. NIHSS, National Institutes of Health Stroke Scale; ONT, onset to needle time; DNT, door to needle time; OCSP, Oxfordshire Community Stroke Project; LACI, lacunar infarct; TACI, total anterior circulation infarct; PACI, partial anterior circulation infarcts; POCI, posterior circulation infarcts; LDL, low-density lipoprotein; INR, international normalized ratio.

**Table 2:** Pharmacological treatments during hospitalization treatments.

	Hemorrhagic transformation	Non- Hemorrhagic transformation	*P* Value
Antiplatelet therapy after admission			
Aspirin 100 mg/d, n(%)	2 (11.1)	144 (38.1)	0.02
Clopidogrel 75 mg/d, n(%)	3 (12.5)	57 (14.5)	0.782
Aspirin 100 mg/d+ Clopidogrel 75 mg/d, n(%)	2 (11.1)	138 (36.5)	0.028
Warfarin, n(%)	0 (0)	16 (4.1%)	0.59
Statins			
Rosuvastatin, n(%)	1 (5.9)	28 (7.5)	0.95
Atorvastatin, n(%)	12 (50.0)	314 (79.3)	0.002
Antihypertensive drugs, n(%)	2 (12.5)	69 (18.6)	0.77
Antidiabetic drugs, n(%)	1 (4.2)	49 (12.4)	0.47

For HT, more patients were found with low level of albumin (15.3% vs. 4.2%, *P* = 0.002), and the incidence of symptomatic hemorrhage was different between two groups (6.2% vs. 1.4%, *P* = 0.03).

In univariate analysis for HT, atrial fibrillation and level of albumin were identified as significant factors (*P* < 0.05). The association of albumin level with HT was carried out by multiple logistic regression after adjusted for all variables with *P* < 0.1. In the multiple logistic regression analysis, low level of serum albumin remained a significant risk factor (OR = 4.369, 95% CI = 1.626–11.742, *P* = 0.003). Besides, atrial fibrillation was also the risk factor of HT after IV rt-PA (OR = 6.888, 95% CI = 2.668–17.780, *P* < 0.001) (see Table 3).

**Table 3:** Binary Regression Analyses Regarding the Occurrence of HT.

	Unadjust OR	95% CI	*P* Value	Adjusted^†^ OR	95% CI	*P* Value
Age, per year	1.038	0.999–1.079	0.056	1.004	0.961–1.050	0.857
Male sex	1.776	0.754–4.184	0.189	—	—	—
NIHSS at baseline, per point	1.062	1.007–1.120	0.027	1.047	0.985–1.113	0.140
ONT, per minutes	0.996	0.990–1.002	0.201	—	—	—
DNT, per minutes	1.003	0.993–1.014	0.551	—	—	—
Hypertension	0.805	0.343–1.889	0.619	—	—	—
Diabetes mellitus	1.035	0.432–2.481	0.939	—	—	—
Atrial fibrillation	7.132	3.005–16.927	<0.001	6.888	2.668–17.780	<0.001*
Coronary artery disease	2.412	0.992–5.861	0.052	1.835	0.681–4.943	0.230
Current smoking	0.683	0.419–1.114	0.127	—	—	—
Previous stroke	0.590	0.197–1.768	0.346	—	—	—
Glucose, per mmol/L	0.999	0.982–1.016	0.918	—	—	—
LDL-cholesterol, per mmol/L	0.653	0.368–1.161	0.147	—	—	—
INR, per unit	0.987	0.741–1.314	0.928	—	—	—
Antiplatelet medicine	0.983	0.283–3.415	0.978	—	—	—
Anticoagulants medicine	0	0	0.999	—	—	—
Level of albumin	4.152	1.726–9.990	0.001	4.369	1.626–11.742	0.003*

*P < 0.05. NIHSS, National Institutes of Health Stroke Scale; ONT, onset to needle time; DNT, door to needle time; LDL, low-density lipoprotein; INR, international normalized ratio; CI indicates confidence interval; OR, odds ratio; HT, hemorrhagic transformation; OCSP, Oxfordshire Community Stroke Project.

^†^Adjusted for all variables with P < 0.1.

## Discussion

According to previously published reports, numbers of factors such as massive cerebral infarction, area of infarction, atrial fibrillation, higher NIHSS, hyperglycemia can increase the risk of ICH after rt-PA administration^6–8^. Elevated globulin level is identified as an independent risk factor of ICH after stroke by involving inflammatory response, such as inflammatory cytokines (IL-1, IL-6, TNFα), matrix metallopeptidase 9 (MMP-9) and positive acute phase reactants synthesized by the liver^9^. The Serum albumin can be useful to measure the nutrition status. Baseline serum albumin measured within 24 hours after stroke onset may not be affected by the acute stress response for the reason of a long half time^10^. Aptaker *et al*.^11^ suggested that the level of serum albumin may relate to the medical complication rate. However, no study has documented the relationship between serum albumin and HT before. Our study indicates that low serum albumin can increase the risk of symptomatic or asymptomatic HT after intravenous thrombolytic therapy, and the effect remained significant even after adjusting for age, Atrial fibrillation, and NIHSS.

Fundamentally, post-stroke HT occurs when BBB permeability increases after vessel reperfusion related to proteolysis, oxidative stress and leukocyte infiltration, especially occurring in the absence of rt-PA or surgery^12–14^. Further rupture of BBB damages the neurovascular unit, which consists of the extracellular matrix, endothelial cells, astrocytes, neurons. Serum albumin, as a specific inhibitor of endothelial cell apoptosis and a major antioxidant defender^3^, can against endogenous and exogenous oxidation. By avidly binding to copper ions, albumin inhibits copper ion-dependent lipid peroxidation at cell membrane^15^. Albumin also transports fatty acids to the post-stroke brain^16^. Because of it neuroprotection function, the rate of HT decreased in normal level of albumin group.

Several studies proved that the serum albumin at admission would be a useful predictor of the functional outcome^4, 5^, low level of serum albumin (<3.2 g/dL) caused poor outcome of AIS^17^. However, in our study, the level of albumin has no effect on clinical outcome at 90 days. But there seem to be a trend of good clinical outcome evaluated by Barthel Index at 7 days in patients with normal level of albumin. For the probably reason that IVT significantly increased the patient’s functional outcome at 90 days, so the adverse consequences of low level of serum albumin obscure. Meanwhile, the small number of patients with hypoproteinemia may produce statistical bias. In experimental studies, albumin can not only reduce hematocrit level but also influence erythrocyte aggregation by increasing low shear viscosity and decreasing erythrocyte sedimentation under no-flow conditions^2^. So it plays a role of blood dilution which increase the cerebral blood flow. Sufficient blood perfusion rescues more neuron in the ischemic penumbra area and reduces harmful injury. Therefore, patients with normal serum albumin levels got a better outcome at 7 days. Previous studies have already shown the safety and efficiency of the high-dose human albumin (0.34–2.05 g/kg) therapy administrated after intravenous rt-PA within 16 hours of stroke onset. The subjects are 3 times more likely to achieve a good outcome than those who received lower-dose albumin^18, 19^. This further demonstrates the neuroprotective effect of albumin in ischemic stroke.

In our study, fewer statins were used in patients with low level of albumin (*p* < 0.05). The probably reason is that more HT were found in low level of albumin group, for the uncertainty of statins for the HT, some clinicians stopped the use of it. Antiplatelet medicine or warfarin were used less in HT patients for fearing aggregation of intracranial hemorrhage after admission. A meta-analysis has reported an association between statin and increased fatality in studies restricted to tPA-treated patients^20^, which together with the increased rates of intracerebral hemorrhage in SPARCL trial (Stroke Prevention by Aggressive Reduction in Cholesterol Levels)^21^. However, in a pilot study, no significant differences were found in the rate of hemorrhagic transformation of any type, nor symptomatic hemorrhagic transformation^22^.

There are some limitations of our study. Because this was a single-center retrospective study, the relatively small sample size and single evaluation index may be not able to represent the whole AIS patients. In addition, we focused on HT rather than symptomatic HT which is a condition well known to be related to poor outcome. Meanwhile, we did not carried out the relationship between albumin levels and function outcomes in ischemic stroke subtypes. These all what we should do a further demonstration.

In conclusion, this study demonstrates that the low serum albumin is an independent predictive factor of HT after IV rt-PA. Further studies are needed to confirm these promising results. The evaluation of serum albumin within 24 h may enable us to identified the risk of HT and future unfavorable prognosis in patients treated with IV rt-PA. Therefore, receiving infusion of albumin for the correction of hypoalbuminemia from the acute stage may be helpful in decrease HT of AIS patients after IV rt-PA.

## Methods

### Patients and ethic statement

An Observation Study of Recanalization Therapy for Acute Ischemic Stroke (STAR) is a registration study in Xuanwu hospital, Capital Medical University. This study was approved by the Research Ethics Committee in Xuanwu hospital, for the recollecting of information. Informed consent was obtained from patients or legal representatives. This study was carried out in accordance with the approved guidelines. Data were collected blinded to the results.

Consecutive AIS patients receiving IV rt-PA thrombolysis from January 1^st^ 2013 to October 31^st^ 2016 were included. All patients were treated with rt-PA within 4.5 h post-onset IV rt-PA (alteplase, 0.9 mg/kg up to a maximum of 90 mg) was used with 10% of the total dosage as a bolus, followed by a 60-min infusion of the remaining dose. The patients were received anti-platelet therapy (aspirin 100 mg/d or clopidogrel 75 mg/d or both). Anticoagulation therapy was performed for the patients with atrial fibrillation, prosthetic cardiac valve or the infarction suspected to be cardioembolism. For those patients with hemorrhagic transformation, the anticoagulation or/and antiplatelet therapy was stopped at once. All the patients received the appropriate rehabilitation guidance according to the clinical condition.

The baseline demographic, clinical and laboratory information collected included hypertension, diabetes mellitus, dyslipidemia, hyperhemocystinemia, coronary artery disease, prior stroke, current smoking, anti-platelet medicine, anti-coagulation medicine, a complete blood count, serum albumin level, glucose, coagulation test, LDL-c, serum albumin levels within 24 h after symptom onset, thrombolysis-related information including Door-to-Needle time (DNT) and Onset-to-Needle time (ONT).

Subjects were categorized into three groups according to serum albumin level: low albumin level (lower than 35 mmol/L), normal albumin level (35–55 mmol/L), high albumin level (above 55 mmol/L)^23, 24^.

### Hemorrhagic transformation and functional outcome

Computed tomography (CT) was performed within 24 hours after IV rt-PA, or performed whenever needed. Magnetic resonance image (MRI) was performed within 7 days after stroke onset. Hemorrhagic transformation was confirmed by brain CT scan or MRI.

Stroke severity was assessed with the National Institute of Health Stroke Score (NIHSS) and the functional status was defined as Barthel Index on admission and during patients’ hospital stay. Modified Rankin Scale (mRS) scores were also evaluated at 7 days and 90 days. These assessments were performed by the experienced neurologists blinded to the illness conditions of patients. Favorable outcomes was defined as mRS < 3. Symptomatic intracranial hemorrhage (sICH) was defined as an ICH on follow-up cerebral imaging combined with clinical deterioration of ≥4 points in NIHSS, or death according to the criteria of the ECASS trial^25^. Follow-up was conducted by telephone or clinical visit.

### Statistical Analysis

Continuous variables are presented as median with interquartile range (IQR) and categorical variables are showed in percentages. Baseline variables on demographical and clinical data were compared by student’s t-test or Wilcoxon-W-test for continuous variables and Chi-square test for categorical variables. To investigate whether level albumin is association with HT in AIS patients, different statistical methods were used. First, we used the logistic regression model. The univariate logistic regression analysis was performed to find variables that were accounted for HT. To adjust for confounding factors with *P* < 0.1, multivariate logistic regression analysis was used to assess any independent factors of HT. All statistical analysis was analyzed by SPSS 21.0. Value of *P* < 0.05 was considered statistically significant.

### Data availability

The datasets generated and analyzed during the current study are available from the corresponding author on reasonable request.

## References

[1] Belayev L, Busto R, Zhao W, Clemens JA, Ginsberg MD (1997). Effect of delayed albumin hemodilution on infarction volume and brain edema after transient middle cerebral artery occlusion in rats. J. Neurosurg..

[2] Reinhart WH, Nagy C (1995). Albumin affects erythrocyte aggregation and sedimentation. Eur J Clin Invest..

[3] Zoellner H (1996). Serum albumin is a specific inhibitor of apoptosis in human endothelial cells. J. Cell Sci..

[4] Dziedzic T, Slowik A, Szczudlik A (2004). Serum albumin level as a predictor of ischemic stroke outcome. Stroke..

[5] Cho YM (2008). Serum albumin at admission for prediction of functional outcome in ischaemic stroke patients. Neurol Sci..

[6] Zhang J, Yang Y, Sun H, Xing Y (2014). Hemorrhagic transformation after cerebral infarction: current concepts and challenges. Ann Transl Med..

[7] Alvarez-Sabin J, Maisterra O, Santamarina E, Kase CS (2013). Factors influencing haemorrhagic transformation in ischaemic stroke. Lancet. Neurol..

[8] Paciaroni M (2008). Early hemorrhagic transformation of brain infarction: rate, predictive factors, and influence on clinical outcome: results of a prospective multicenter study. Stroke..

[9] Xing Y (2014). Increased globulin and its association with hemorrhagic transformation in patients receiving intra-arterial thrombolysis therapy. Neurosci bull..

[10] Hall CA (1982). Nutritional assessment. New Eng J Med..

[11] Aptaker RL, Roth EJ, Reichhardt G, Duerden ME, Levy CE (1994). Serum albumin level as a predictor of geriatric stroke rehabilitation outcome. Arch Phys Med Rehabil..

[12] Jickling GC (2014). Hemorrhagic transformation after ischemic stroke in animals and humans. J Cereb Blood Flow Metab..

[13] Nour M, Scalzo F, Liebeskind DS (2013). Ischemia-reperfusion injury in stroke. Interv Neurol..

[14] Sumii T, Lo EH (2002). Involvement of matrix metalloproteinase in thrombolysis-associated hemorrhagic transformation after embolic focal ischemia in rats. Stroke..

[15] Hallenbeck JM (1986). Polymorphonuclear leukocyte accumulation in brain regions with low blood flow during the early postischemic period. Stroke..

[16] Rodriguez de Turco EB (2002). Systemic fatty acid responses to transient focal cerebral ischemia: influence of neuroprotectant therapy with human albumin. J. Neurochem..

[17] Babu MS (2013). Serum albumin levels in ischemic stroke and its subtypes: correlation with clinical outcome. Nutrition..

[18] Ginsberg MD, Hill MD, Palesch YY, Ryckborst KJ, Tamariz D (2006). The ALIAS Pilot Trial: a dose-escalation and safety study of albumin therapy for acute ischemic stroke–I: Physiological responses and safety results. Stroke..

[19] Palesch YY, Hill MD, Ryckborst KJ, Tamariz D, Ginsberg MD (2006). The ALIAS Pilot Trial: a dose-escalation and safety study of albumin therapy for acute ischemic stroke–II: neurologic outcome and efficacy analysis. Stroke..

[20] Ní CD (2013). Statin therapy and outcome after ischemic stroke: systematic review and meta-analysis of observational studies and randomized trials. Stroke..

[21] Goldstein LB (2009). Statin treatment and stroke outcome in the Stroke Prevention by Aggressive Reduction in Cholesterol Levels (SPARCL) trial. Stroke.

[22] Montaner J (2016). Combination of Thrombolysis and Statins in Acute Stroke Is Safe: Results of the STARS Randomized Trial (Stroke Treatment With Acute Reperfusion and Simvastatin). Stroke..

[23] Dziedzic T (2006). Serum albumin level and nosocomial pneumonia in stroke patients. Eur J Neuro..

[24] Dziedzic T, Pera J, Slowik A, Gryz-Kurek EA, Szczudlik A (2007). Hypoalbuminemia in acute ischemic stroke patients: frequency and correlates. Eur J Clin Nutr..

[25] Fiorelli M (1999). Hemorrhagic transformation within 36 hours of a cerebral infarct: relationships with early clinical deterioration and 3-month outcome in the European Cooperative Acute Stroke Study I (ECASS I) cohort. Stroke..

